# A novel autophagy‐related lncRNA prognostic risk model for breast cancer

**DOI:** 10.1111/jcmm.15980

**Published:** 2020-11-20

**Authors:** Xiaoying Li, Feng Jin, Yang Li

**Affiliations:** ^1^ Department of Breast Surgery The First Affiliated Hospital of China Medical University Shenyang China; ^2^ Department of Cell Biology Key Laboratory of Cell Biology Ministry of Public Health, and Key Laboratory of Medical Cell Biology Ministry of Education China Medical University Shenyang China

**Keywords:** autophagy, breast cancer, long non‐coding RNAs (lncRNAs), prognosis, risk model

## Abstract

Long non‐coding RNAs (lncRNAs) are well known as crucial regulators to breast cancer development and are implicated in controlling autophagy. LncRNAs are also emerging as valuable prognostic factors for breast cancer patients. It is critical to identify autophagy‐related lncRNAs with prognostic value in breast cancer. In this study, we identified autophagy‐related lncRNAs in breast cancer by constructing a co‐expression network of autophagy‐related mRNAs‐lncRNAs from The Cancer Genome Atlas (TCGA). We evaluated the prognostic value of these autophagy‐related lncRNAs by univariate and multivariate Cox proportional hazards analyses and eventually obtained a prognostic risk model consisting of 11 autophagy‐related lncRNAs (U62317.4, LINC01016, LINC02166, C6orf99, LINC00992, BAIAP2‐DT, AC245297.3, AC090912.1, Z68871.1, LINC00578 and LINC01871). The risk model was further validated as a novel independent prognostic factor for breast cancer patients based on the calculated risk score by Kaplan‐Meier analysis, univariate and multivariate Cox regression analyses and time‐dependent receiver operating characteristic (ROC) curve analysis. Moreover, based on the risk model, the low‐risk and high‐risk groups displayed different autophagy and oncogenic statues by principal component analysis (PCA) and Gene Set Enrichment Analysis (GSEA) functional annotation. Taken together, these findings suggested that the risk model of the 11 autophagy‐related lncRNAs has significant prognostic value for breast cancer and might be autophagy‐related therapeutic targets in clinical practice.

## 1 INTRODUCTION

Breast cancer is the most frequent malignancy and the leading cause of cancer‐associated mortality in women worldwide.^1^, ^2^ In clinical practice, individual specific targeted therapy has attracted more and more attention. Thus, exploring potential prognostic biomarkers and promising targets is regarded to be an essential step to achieve this goal.

Autophagy is a highly conserved process to maintain cellular homeostasis by lysosomal degradation system.^3^ Autophagy plays a crucial role in many physiological processes and various pathological events, including stress and starvation adaptation, metabolism, inflammation, neurodegenerative disorders and cancer.^4^, ^5^, ^6^, ^7^ Over the past few years, an increasing number of studies have indicated that autophagy participates in the development and progression of breast cancer.^8^, ^9^ Therefore, identifying key regulators of autophagy is of great importance for both theoretical basis and clinical practice.

Long non‐coding RNAs (lncRNAs) are a series of transcript RNAs longer than 200 nucleotides without the capacity of protein‐coding.^10^ LncRNAs are considered as one of the most sensitive and specific cancer biomarkers, which participate in the development and progression of various cancers at different levels including epigenetic, transcriptional and post‐transcriptional regulation.^11^, ^12^, ^13^, ^14^ Moreover, accumulating studies have suggested that lncRNAs promote cancer progression and predict worse prognosis in numerous cancers via regulating autophagy.^15^, ^16^, ^17^ Therefore, it is valuable to identify key lncRNAs closely related to autophagy and prognosis in breast cancer.

In the present study, we analysed a data set of lncRNA expression in breast cancers from The Cancer Genome Atlas (TCGA) and screened out autophagy‐related lncRNAs with prognostic value. We identified an eleven autophagy‐related lncRNA signature with the potential to predict the survival prognosis of breast cancer patients.

## 2 METHODS AND MATERIALS

### 2.1 Patient data sets

Breast cancer patients with clinical information and pathology records were obtained from the TCGA (https://cancergenome.nih.gov/). Normalize gene expression was performed by the edgeR package. In this study, a total of 1053 TCGA female breast cancer patients with lncRNA expression profiles were used. Among them, 986 patients with complete follow‐up information and survival time ≥ 30 days and 539 patients with complete clinicopathological data were selected into subsequent analyses. The clinical features are detailed in Table 1.

**Table 1:** Clinical pathological parameters of patients with breast cancer

Feature	N (539)	%
Age (years)		
>60	227	42.1
≤60	312	57.9
T classification		
T1 (<2 cm)	147	27.3
T2 (2‐5 cm)	323	59.9
T3 (≥5 cm)	55	10.2
T4 (chest wall and/or skin invasion)	14	2.6
N classification (pN)		
N0 (no metastasis)	259	48.1
N1 (1‐3 metastasis)	178	33
N2 (4‐9 metastasis)	64	11.9
N3 (≥10 metastasis)	38	7
M classification		
M0 (no distant metastasis)	528	98
M1 (distant metastasis)	11	2
TNM stage		
I	96	17.8
II	318	59
III	114	21.2
IV	11	2
ER		
Negative	127	23.6
Positive	412	76.4
PR		
Negative	175	32.5
Positive	364	67.5
HER2		
Negative	440	81.6
Positive	99	18.4
Molecular subtypes		
HER2 amplification	92	17.1
Luminal A/B	419	77.7
TNBC	28	5.2

Note

T, tumour size; N, lymph node; M, distant metastasis; TNM stage, according to AJCC 8th classification; TNBC, triple‐negative breast cancer.

### 2.2 Identification of autophagy‐related lncRNAs in breast cancer

A total of 395 autophagy‐related encoding genes (mRNAs) were extracted from the Molecular Signatures Database of Gene Set Enrichment Analysis (GSEA: M27935, M6328 and M10281). Finally, 912 autophagy‐related lncRNAs were identified by constructing autophagy‐related mRNA‐lncRNA co‐expression network according to the criteria of |Correlation Coefficient| > 0.4 and *P* < .001 by Pearson correlation analysis using the Limma R package.^18^

### 2.3 Identification of autophagy‐related lncRNA prognostic signatures for breast cancer

To identify autophagy‐related lncRNAs associated with survival, we performed univariate Cox proportional hazards analysis according to the criteria of *P* < .01. Subsequently, multivariate Cox analysis was conducted to construct the optimal prognostic risk model based on the Akaike information criterion (AIC = 1444.62), using the survival R package. Based on the following formula, the risk score for each patient was calculated.

$$\text{Risk score}=\text{coef}\left(\text{lncRNA}1\right)\times \text{expr}\left(\text{lncRNA1}\right)+\text{coef}\left(\text{lncRNA2}\right)\times \text{expr}\left(\text{lncRNA2}\right)+\ldots +\;\text{coef}\left(\text{lncRNAn}\right)\times \text{expr}\left(\text{lncRNAn}\right).$$

coef (lncRNAn) was defined as the coefficient of lncRNAs correlated with survival.

expr (lncRNAn) was defined as the expression of lncRNAs.

Based on the median risk score, breast cancer patients in the TCGA were divided into a high‐risk group and a low‐risk group. Kaplan‐Meier survival analysis was performed to estimate the survival difference between the two groups by using the survival and survminer R packages.

### 2.4 Independent prognostic analysis and ROC curve plotting

To assess the relationship of survival prognosis with clinicopathological factors and risk score, we performed univariate and multivariate Cox regression analyses using the Survival R package. Time‐dependent receiver operating characteristic (ROC) curves were drew to estimate the predictive accuracy for survival time by different clinical pathological factors and risk score using the survivalROC R package.

### 2.5 Statistical analysis

All statistical analyses were performed using R software (version 3.6.2). A co‐expression network of the 11 autophagy‐related lncRNAs‐mRNAs with prognostic value was established and visualized using Cytoscape and Sankey diagram. The correlation between 11 autophagy‐related lncRNA expressions and clinicopathological factors was analysed by ggpubr R package. Principal component analysis (PCA) was performed for effective dimension reduction, pattern recognition and exploratory visualization of high‐dimensional data of the whole‐genome, 395 autophagy‐related encoding genes and the risk model of the 11 autophagy‐related lncRNA expression profiles, respectively.^19^, ^20^ Gene Set Enrichment Analysis (GSEA) was used for functional annotation. GSEA (https://www.gsea‐msigdb.org/gsea/index.jsp) is a powerful analytical approach for interpreting genome‐wide expression profiles.^21^ GSEA focuses on gene sets rather than just high scoring genes, which can detect biological processes such as several cancer‐related pathways, metabolic pathways, transcriptional programmes and stress responses. GSEA tends to be more reproducible and more interpretable to analyse molecular profiling data. Two‐tailed *P* < .05 was considered statistically significant.

## 3 RESULTS

### 3.1 Identification of autophagy‐related lncRNAs with significant prognostic value in breast cancer

**Figure 1:**
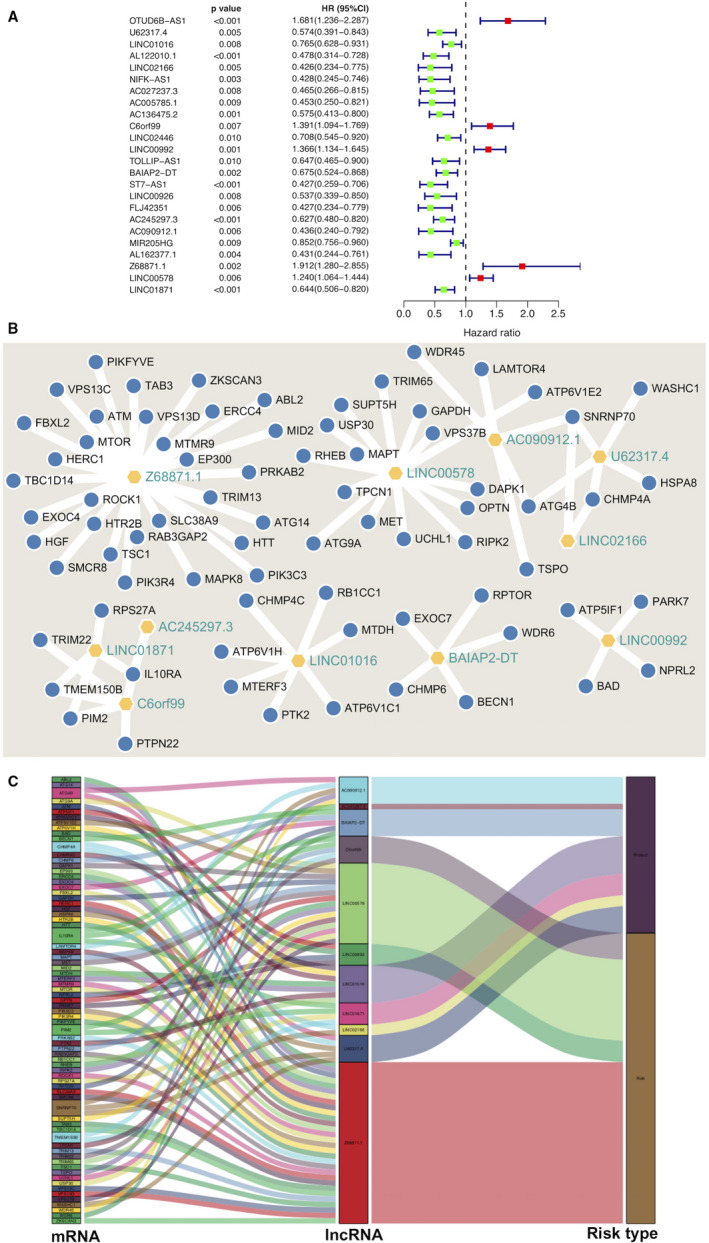
Identification of autophagy‐related lncRNAs with significant prognostic value in breast cancer. A, The forest showed the HR (95% CI) and p‐value of selected lncRNAs by univariate Cox proportional hazards analysis. B and C, A co‐expression network of the 11 autophagy‐related lncRNAs‐mRNAs with prognostic value was constructed and visualized using Cytoscape and Sankey diagram

**Figure 2:**
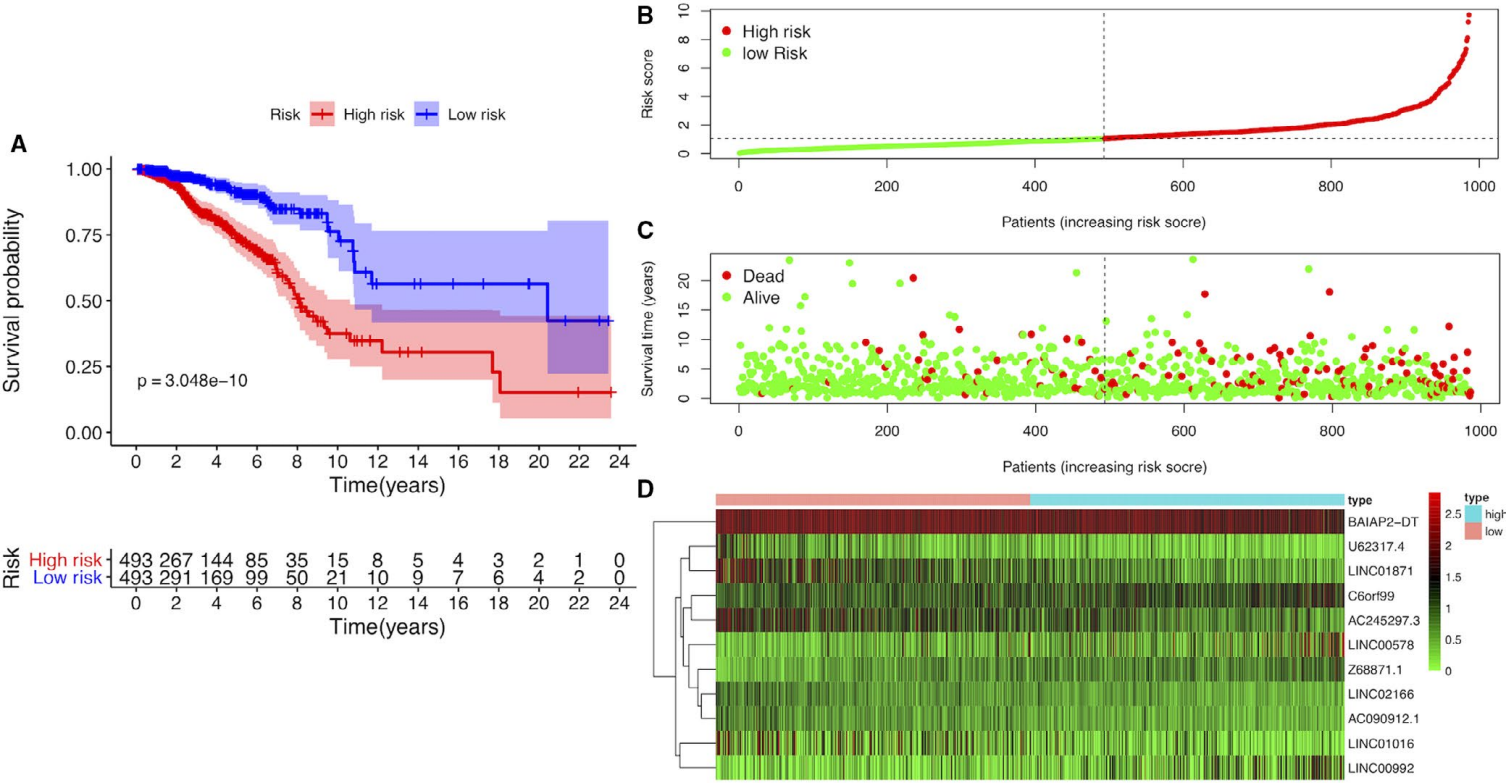
The prognostic value of the risk model of the 11 autophagy‐related lncRNAs in the TCGA cohort. A, Kaplan‐Meier survival analysis of the high‐risk and low‐risk groups based on the risk model and median risk score. B, The risk curve based on the risk score of each sample. C, The scatterplot based on the survival status of each sample. The green and red dots represent survival and death, respectively. D, The heatmap displayed the expression levels of autophagy‐related lncRNAs in the high‐risk and low‐risk groups

A total of 912 autophagy‐related lncRNAs were obtained by constructing the co‐expression networks with 395 autophagy‐related encoding genes (mRNAs). Among them, 24 autophagy‐related lncRNAs were significantly associated with the survival of breast cancer patients from the TCGA (*P* < .01) by Cox proportional hazards analysis, including 19 lncRNAs with low risk (hazard ration (HR)<1) and 5 lncRNAs with high risk (hazard ration (HR)>1) (Figure 1A). Subsequently, multivariate Cox analysis further screened 11 lncRNAs from the above 24 autophagy‐related lncRNAs with prognostic significance, namely, U62317.4, LINC01016, LINC02166, C6orf99, LINC00992, BAIAP2‐DT, AC245297.3, AC090912.1, Z68871.1, LINC00578 and LINC01871 (Table 2). These 11 lncRNAs were constituted into the optimal prognostic risk model of autophagy‐related lncRNAs. As shown in Figure 1B‐C, A visualization co‐expression network of the 11 autophagy‐related lncRNAs‐mRNAs with prognostic value was established. On the basis of the risk score formula and the calculated median risk score, breast cancer patients were divided into a high‐risk group and a low‐risk group. Kaplan‐Meier survival analysis showed that the high‐risk group displayed worse overall survival (OS) than the low‐risk group (*P* = 3.048E−10) (Figure 2A), indicating that the risk score has prognostic value. The risk curve and scatterplot were applied to illustrate the risk score and the relevant survival statuses of breast cancer patients. The results indicated that the mortality occurrence depended on the risk score (Figure 2B‐C). The heatmap of these 11 autophagy‐related lncRNA expressions in breast cancer samples showed that C6orf99, LINC00992, Z68871.1 and LINC00578 were highly expressed in the high‐risk group, while U62317.4, LINC01016, LINC02166, BAIAP2‐DT, AC245297.3, AC090912.1 and LINC01871 were up‐regulated in the low‐risk group (Figure 2D). Therefore, these above studies identified 11 autophagy‐related lncRNAs with prognostic significance for breast cancer.

**Table 2:** The risk model of 11 autophagy‐related lncRNAs with prognostic value for breast cancer by multivariate Cox proportional hazards analysis

LncRNA	Coef	HR	HR.95L	HR.95H	*P*‐value	Risk
U62317.4	−0.308	0.735	0.488	1.106	.140	low
LINC01016	−0.173	0.842	0.678	1.045	.119	low
LINC02166	−0.596	0.551	0.292	1.041	.066	low
C6orf99	0.284	1.329	1.017	1.738	.038	high
LINC00992	0.188	1.207	0.988	1.473	.065	high
BAIAP2‐DT	−0.385	0.680	0.525	0.882	.004	low
AC245297.3	−0.381	0.683	0.520	0.897	.006	low
AC090912.1	−0.475	0.622	0.342	1.131	.119	low
Z68871.1	0.573	1.774	1.133	2.778	.012	high
LINC00578	0.146	1.158	0.979	1.369	.087	high
LINC01871	−0.417	0.659	0.495	0.878	.004	low

Note

coef: the coefficient of lncRNAs correlated with survival; HR: hazard ratio; HR.95L: low 95% CI of HR; HR.95H: high 95% CI of HR.

### 3.2 Evaluation of the risk model of 11 autophagy‐related lncRNAs as an independent prognostic factor for breast cancer patients

**Figure 3:**
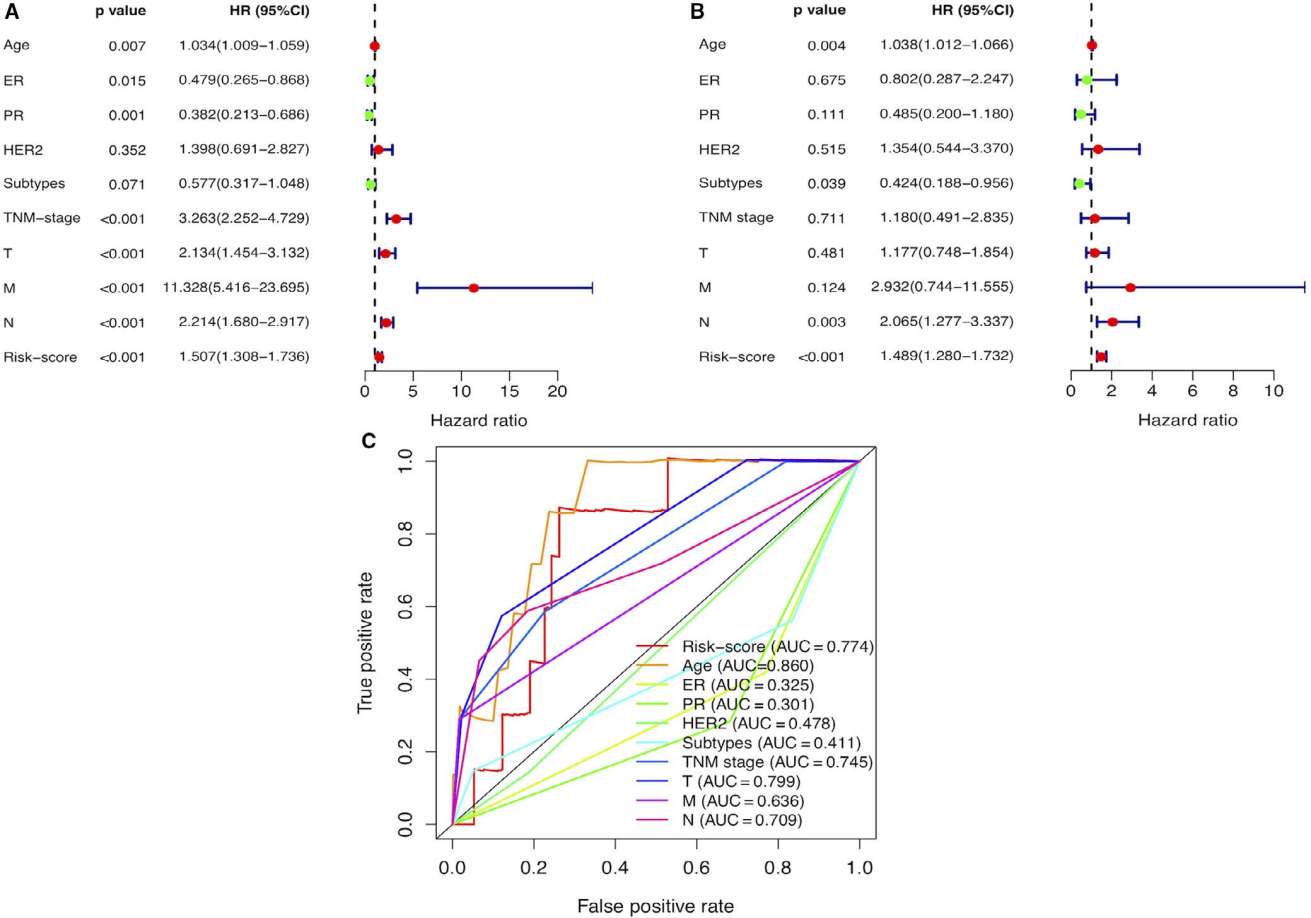
Assessment of the prognostic risk model of the 11 autophagy‐related lncRNAs in breast cancer. A, The univariate and B, multivariate Cox regression analysis of risk model score and clinical features regarding prognostic value. C. The AUC for risk model score and clinical features according to the ROC curves. Clinical features: age, ER, PR, HER2, subtypes (molecular subtypes), TNM stage, T (tumour size), N (lymph node metastasis) and M (distant metastasis)

To evaluate whether the risk model of the above 11 autophagy‐related lncRNAs is an independent prognostic factor for breast cancer, univariate and multivariate Cox regression analyses were conducted. The hazard ratio (HR) of the risk score and 95% CI were 1.507 and 1.308‐1.736 (*P* < .001) in univariate Cox regression analysis (Figure 3A), and 1.489 and 1.280‐1.732 (*P* < .001) in multivariate Cox regression analysis (Figure 3B), respectively, suggesting that the risk model of the 11 autophagy‐related lncRNAs is the most significant prognostic factor for breast cancer, independent of clinicopathological parameters such as oestrogen receptor (ER) expression; progesterone receptor (PR) expression; human epidermal growth factor receptor (HER2) expression; tumour, node, metastasis (TNM) stage; tumour size; lymph node metastasis; and distant metastasis. To assess the predictive sensitivity and specificity of the risk score on the prognosis of breast cancer patients, the area under the ROC curve (AUC) of the risk score was calculated. The AUC of the risk score was 0.774, similar to the AUC of tumour size, followed by the AUC of age and more than the AUCs of other clinicopathological factors (Figure 3C), indicating that the prognostic risk model of the 11 autophagy‐related lncRNAs for breast cancer is considerably reliable. Taken together, these all indicated that the risk model of the 11 autophagy‐related lncRNAs is significant independent prognostic factor for breast cancer patients.

### 3.3 Correlation of the expression of the 11 autophagy‐related lncRNAs with clinicopathological factors

**Figure 4:**
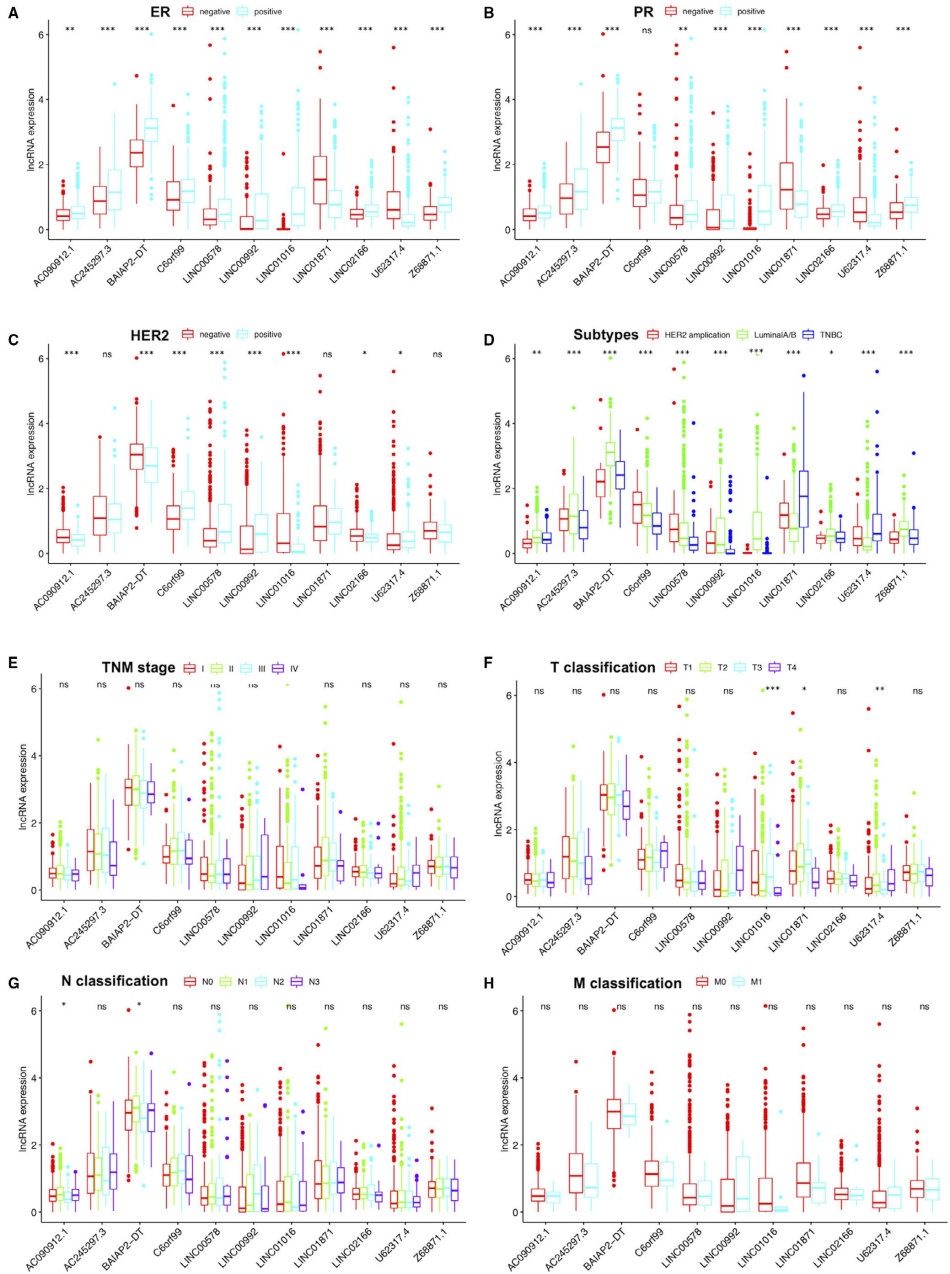
The correlation of the expression of the 11 autophagy‐related lncRNAs with clinicopathological factors. A, ER expression. B, PR expression. C, HER2 expression. D, Subtypes (luminal A/B; HER2 amplification; TNBC: triple‐negative breast cancer). E, TNM stage. F, Tumour size (T1: <2cm; T2: ≥2cm and < 5cm; T3: ≥5cm; T4: invasion of chest wall and/or skin). G, N classification (N0: no lymph node metastasis; N1: 1‐3 lymph node metastasis; N2: 4‐9 lymph node metastasis; N3: ≥10 lymph node metastasis). H, M classification (M0: no distant metastasis; M1: distant metastasis). ns: no statistical significance, *P < .05, **P < .01, ***P < .001, ****P < .0001

To further assess whether the 11 autophagy‐related lncRNAs participated in the development of breast cancer, we investigated the association of the expression of the 11 autophagy‐related lncRNAs with clinicopathological factors. There were remarkably correlations between a majority of the 11 autophagy‐related lncRNAs and ER expression, PR expression, HER2 expression and molecular subtypes, as shown in Figure 4.

### 3.4 Different autophagy statuses in the low‐risk and high‐risk groups

**Figure 5:**
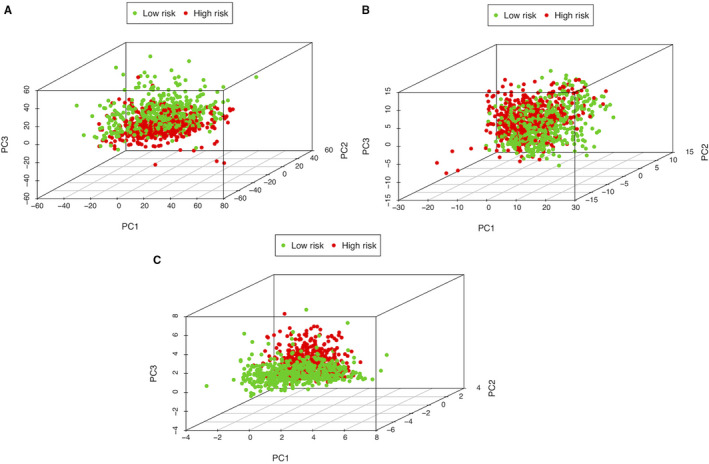
The low‐risk and high‐risk groups displayed different autophagy statuses. A‐C, Principal component analysis (PCA) between low‐risk and high‐risk groups based on the whole‐genome, autophagy‐related encoding genes and the risk model of the 11 autophagy‐related lncRNA expression profiles.

**Figure 6:**
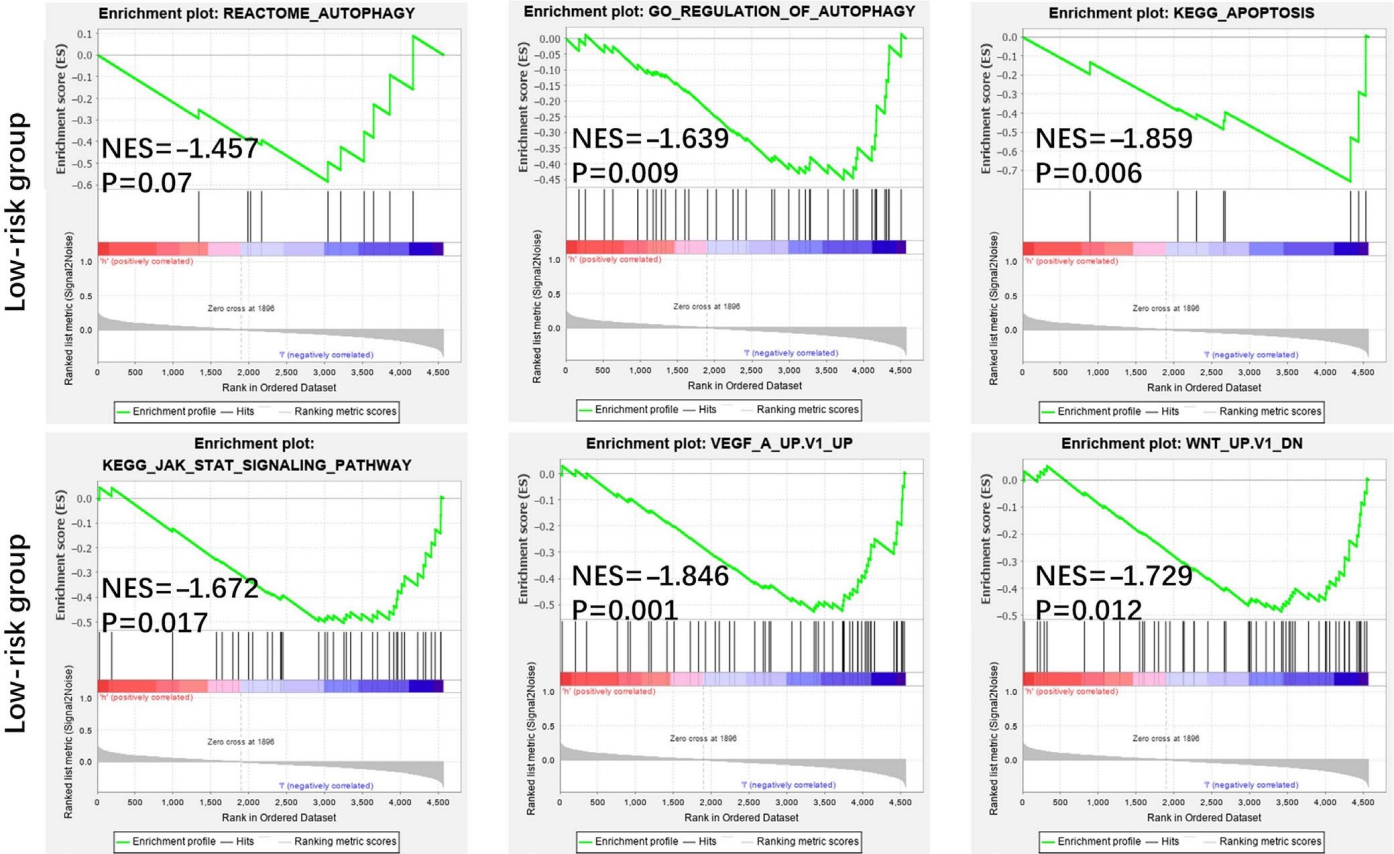
Functional enrichment analysis based on the risk model of the 11 autophagy‐related lncRNAs by GSEA. Significantly enriched autophagy of Gene Ontology (GO), KEGG pathways and oncogenic signatures in the low‐risk group

PCA was performed to compare the difference between low‐risk and high‐risk groups based on the risk model of the 11 autophagy‐related lncRNAs, 395 autophagy‐related encoding genes and the whole‐genome expression profiles from the TCGA, respectively (Figure 5). The results showed that the low‐risk and high‐risk groups were distributed in distinct directions, prior to the other two patterns, suggesting that the risk model could divide breast cancer patients into two parts and that the autophagy status of breast cancer patients in the high‐risk group differed from those in the low‐risk group. Functional annotation was further conducted using GSEA, and the results displayed that the differentially expressed genes between the high‐risk and low‐risk groups based on the risk model of the 11 autophagy‐related genes were enrichment in autophagy processes and oncogenic signatures (Figure 6).

Above these, it all indicated that the low‐risk and high‐risk groups showed different autophagy and oncogenic statuses.

## 4 DISCUSSION

In the field of clinical treatment, although the overall survival of breast cancer patients has gained great improvements, metastasis and recurrence of breast cancer have constantly grown, which were the major causes of breast cancer mortality. Considerable evidence has revealed that the role of autophagy in the development of cancer is a double‐edged sword. That is, autophagy may serve as either a pro‐survival or pro‐death mechanism under different circumstances.^22^, ^23^ Consistent with the theory, autophagy functioned dual roles in breast cancer. Autophagy induced the recurrence of metastatic breast cancer by elevating the survival of dormant breast cancer cells.^8^ Cytostatic autophagy suppressed the proliferation and metastasis of triple‐negative breast cancer cells.^9^ Furthermore, numerous researches indicated the crucial role of lncRNAs in autophagy‐inducing progression or inhibition of various cancers, such as hepatocellular carcinoma, lung cancer and breast cancer.^24^, ^25^, ^26^ Based on the above, it led us to find potential specific lncRNAs associated with autophagy and the survival prognosis. In this study, we identified the risk model of the 11 autophagy‐related lncRNAs as an independent prognostic factor for breast cancer. So far, among these 11 autophagy‐related lncRNAs, only LINC01016 and LINC00578 have been studied in breast cancer or other cancers. LINC01016‐miR‐302a‐3p/miR‐3130‐3p/NFYA/SATB1 axis plays a crucial role in the occurrence of endometrial cancer.^27^In addition, LINC01016 is a direct target of ERα, associated with survival prognosis of breast cancer.^28^ It has been reported that LINC00578 is associated with OS in pancreatic cancer and lung adenocarcinoma.^29^, ^30^ In accordance with our results, LINC01016 was low‐risk autophagy‐related lncRNA and LINC00578 was high‐risk autophagy‐related lncRNA, both with prognostic value in breast cancer patients. Breast cancer is a highly heterogeneous tumour and has been divided into distinct molecular subtypes as follows: luminal A/B (ER and/or PR positive), HER2 enriched (HER2 positive) and triple‐negative breast cancer (ER, PR and HER2 negative).^31^, ^32^ Distinct ER, PR and HER2 statuses and molecular subtypes suggested different biological processes of breast cancer and survival outcomes.^33^, ^34^ The significant correlations of most of the 11 autophagy‐related lncRNAs with ER expression, PR expression, HER2 expression and molecular subtypes indicated that the 11 autophagy‐related lncRNAs might be involved in regulating ER, PR and HER2 gene expression, further affecting molecular subtype formation. More interestingly, most of the 11 autophagy‐related lncRNAs had no significant association with tumour size, lymph node status and TNM stage, implying that there is no close relationship between the timing of breast cancer diagnosis and the risk model of the 11 autophagy‐related lncRNAs. All above results indicated that the risk model strongly links to the intrinsic biological characteristics of distinct subtypes of breast cancer. Our results also showed that the risk model of the 11 autophagy‐related lncRNAs had superior prognostic value to other clinicopathological factors. The results of PCA and GSEA indicated that the significant difference in OS between the high‐risk and low‐risk groups might result from different autophagy and oncogenic statuses induced by the risk model. These results also suggested that the risk model of the 11 autophagy‐related lncRNAs represented more significant autophagy characteristics than autophagy‐related genes. Taken together, these results suggested that the prognostic risk model of the 11 autophagy‐related lncRNAs might be a feasible independent prognostic factor for breast cancer in clinical practice.

To date, the focus of precision genomic medicine is to find out accurate specific predictive factors for survival prognosis from large medical data sets with clinical outcomes.^35^ Thus, there have been some researches aiming to explore autophagy‐related prognostic factors using bioinformatics analysis in recent years. Over the past year, three distinct prognostic risk models of autophagy‐related encoding genes in breast cancer have been established based on TCGA database by using different screening criteria and statistics methods.^36^, ^37^, ^38^ Meanwhile, due to the crucial function of lncRNAs in autophagy, autophagy‐related lncRNAs in cancer also arouse more attention.^15^, ^16^, ^17^ Recently, the prognostic risk models of autophagy‐related lncRNAs were constructed in several cancers including bladder urothelial carcinoma and glioma.^39^, ^40^ However, the role of autophagy‐related lncRNAs in prognosis of breast cancer remains unclear. We therefore conducted this research and found out a novel eleven autophagy‐related lncRNA prognostic risk model, which may assist clinicians in making individual effective therapeutic decisions.

However, our study has some limitations. First, we applied traditional statistical analyses to build and evaluate the prognostic risk model of 11 autophagy‐related lncRNAs. Although the methods have been utilized and validated in many researches, it is critical to improve our further studies with more advanced methodologies and technologies in the future. To further verify our bioinformatics prediction results, in‐depth studies on the 11 autophagy‐related lncRNAs, including functional experiments and molecular mechanisms, are needed.

## 5 CONCLUSION

In conclusion, we identified a novel autophagy‐related prognostic risk model consisting of 11 lncRNAs (U62317.4, LINC01016, LINC02166, C6orf99, LINC00992, BAIAP2‐DT, AC245297.3, AC090912.1, Z68871.1, LINC00578 and LINC01871) in breast cancer. In the future, with prospective validation, the 11 autophagy‐related lncRNAs signature may improve predictive accuracy and guide individualized therapy for breast cancer patients.

## CONFLICTS OF INTEREST

The authors declare no conflicts of interest.

## AUTHOR CONTRIBUTION

**Xiaoying Li:** Conceptualization (equal); Data curation (equal); Formal analysis (lead); Writing‐original draft (lead); Writing‐review & editing (equal). **Feng Jin:** Conceptualization (equal); Funding acquisition (equal); Supervision (lead); Writing‐review & editing (lead). **Yang Li:** Conceptualization (equal); Funding acquisition (lead); Supervision (lead); Writing‐review & editing (lead).

## Data Availability

All data utilized in this study are included in this article, and all data supporting the findings of this study are available on reasonable request from the corresponding author.
